# Strain Effects on the Electronic and Thermoelectric Properties of n(PbTe)-m(Bi_2_Te_3_) System Compounds

**DOI:** 10.3390/ma14154086

**Published:** 2021-07-22

**Authors:** Weiliang Ma, Marie-Christine Record, Jing Tian, Pascal Boulet

**Affiliations:** 1 CNRS, IM2NP, Aix-Marseille University, University of Toulon, 13013 Marseille, France; weiliang.ma@etu.univ-amu.fr (W.M.); jing.tian@etu.univ-amu.fr (J.T.); 2 CNRS, MADIREL, Aix-Marseille University, 13013 Marseille, France; pascal.boulet@univ-amu.fr

**Keywords:** thermoelectricity, biaxial tensile and compressive strains, density functional theory, QTAIM

## Abstract

Owing to their low lattice thermal conductivity, many compounds of the n(PbTe)-m(Bi${}_{2}$Te${}_{3}$) homologous series have been reported in the literature with thermoelectric (TE) properties that still need improvement. For this purpose, in this work, we have implemented the band engineering approach by applying biaxial tensile and compressive strains using the density functional theory (DFT) on various compounds of this series, namely Bi${}_{2}$Te${}_{3}$, PbBi${}_{2}$Te${}_{4}$, PbBi${}_{4}$Te${}_{7}$ and Pb${}_{2}$Bi${}_{2}$Te${}_{5}$. All the fully relaxed Bi${}_{2}$Te${}_{3}$, PbBi${}_{2}$Te${}_{4}$, PbBi${}_{4}$Te${}_{7}$ and Pb${}_{2}$Bi${}_{2}$Te${}_{5}$ compounds are narrow band-gap semiconductors. When applying strains, a semiconductor-to-metal transition occurs for all the compounds. Within the range of open-gap, the electrical conductivity decreases as the compressive strain increases. We also found that compressive strains cause larger Seebeck coefficients than tensile ones, with the maximum Seebeck coefficient being located at −2%, −6%, −3% and 0% strain for *p*-type Bi${}_{2}$Te${}_{3}$, PbBi${}_{2}$Te${}_{4}$, PbBi${}_{4}$Te${}_{7}$ and Pb${}_{2}$Bi${}_{2}$Te${}_{5}$, respectively. The use of the quantum theory of atoms in molecules (QTAIM) as a complementary tool has shown that the van der Waals interactions located between the structure slabs evolve with strains as well as the topological properties of Bi${}_{2}$Te${}_{3}$ and PbBi${}_{2}$Te${}_{4}$. This study shows that the TE performance of the n(PbTe)-m(Bi${}_{2}$Te${}_{3}$) compounds is modified under strains.

## 1. Introduction

Energy production and environment protection have become two of the most critical current issues, as fossil fuels constitute a finite source of energy, and at the same time their consumption drastically affects the climate patterns. Only a fraction of the energy released by the burning of fossil fuels is converted into mechanical energy or electricity, whereas most of that energy is released as heat. Therefore, policy makers and scientists alike are exploring new routes to provide green, clean and efficient energy. Thermoelectric generators (TEGs) are solid-state devices that convert a heat flux directly into electrical power [1]. The heat-to-electricity conversion exploits the Seebeck effect. A thermoelectric (TE) converter is constituted of *n*-type and *p*-type materials, and the absence of moving or mechanical parts allows them to function for a substantially long time without the need for repair. In addition, they are extremely quiet, reliable and scalable, making them ideal for small distributing power generation. One of the main drawbacks of the TE converters is the low conversion efficiency of their constitutive TE materials [2].

In the past decades, many TE materials systems have been studied, such as Half-Heusler alloys [3,4,5,6], chalcogenides [7,8,9,10], skutterudites [11,12,13,14], clathrates [15,16,17,18] and zintl phases [19,20,21,22]. The performance of TE materials is determined by the dimensionless figure of merit $\mathrm{zT}=S^{2}\sigma /\left(\kappa_{e}+\kappa_{l}\right)$, where *S* is the Seebeck coefficient, σ is the electrical conductivity and $\kappa_{e}$ and $\kappa_{l}$ are the electronic part and lattice part of thermal conductivity. High TE efficiency needs high *S* and σ and low thermal conductivity, κ. Improving TE performance has been a big challenge. To meet this challenge, various approaches that can be classified into two categories, namely band engineering and phonon engineering, have been used. Through constructing superlattice structures or creating nanostructures, the phonon-boundary scattering can be enhanced, resulting in an extremely low thermal conductivity [23,24]. On the other hand, the TE properties can be boosted via enhancing effective density of states (DOS) by band convergence near the Fermi level [2,25,26] or changing the forbidden bandwidth by strains [26]. In the present work, tensile and compressive biaxial strains have been applied to Bi${}_{2}$Te${}_{3}$, PbBi${}_{2}$Te${}_{4}$, PbBi${}_{4}$Te${}_{7}$ and Pb${}_{2}$Bi${}_{2}$Te${}_{5}$ to investigate their effects on the thermoelectric and bonding properties of these compounds. The last section is devoted to the investigation of the topological insulator properties of Bi${}_{2}$Te${}_{3}$ and PbBi${}_{2}$Te${}_{4}$ under peculiar strains.

## 2. Methods and Computational Details

The calculations of structural, electronic and thermoelectric properties have been performed within the frame of density functional theory using the all-electron FP-LAPW approach with the local orbital method, as implemented in WIEN2K (version 19.1, 2019, Technology University of Vienna, Vienna, Austria) [27]. Several exchange-correlation functionals have been used, the details of which will be mentioned when appropriate in the results section. Although hybrid exchange-correlation functionals usually yield excellent results compared with experimental ones regarding band-gap energies, as exemplified in [28,29], the large number of calculations performed in the present work preclude the use of such time-consuming functionals. Therefore, the results presented in this work have been obtained with pure, density-based functionals. For structural optimizations, the Brillouin zone has been sampled with the k-meshes 8 × 8 × 8, 8 × 8 × 8, 12 × 12 × 2 and 12 × 12 × 2, for Bi${}_{2}$Te${}_{3}$, PbBi${}_{2}$Te${}_{4}$, PbBi${}_{4}$Te${}_{7}$ and Pb${}_{2}$Bi${}_{2}$Te${}_{5}$, respectively. For the subsequent convergence of the self-consistent energy, the Monkhorst-Pack k-meshes have been set as 16 × 16 × 16, 16 × 16 × 16, 18 × 18 × 4 and 18 × 18 × 2. The valence electrons for Pb, Bi and Te have been taken as 5d${}^{10}$6s${}^{2}$6p${}^{2}$, 5d${}^{10}$6s${}^{2}$6p${}^{3}$ and 4d${}^{10}$5s${}^{2}$5p${}^{6}$. The total energy and atomic forces’ convergence thresholds have been defined as 0.136 meV and 0.257 meV/Å, respectively. The RmtKmax value has been set to 9.0 and the Radius of Muffin Tin (RMT) used for both Bi and Te atoms in this study has been set to 2.5 Å. The structure and charge density calculated above are used to analyze the topological properties within QTAIM theory [30]. The transport properties of the compounds with and without strain have been calculated with the BoltzTraP2 code [31] based on the use of a full band’s structure in the Brillouin zone. The sampling, which is important in transport calculation, has been performed with a very dense k-mesh of 36 × 36 × 36, 36 × 36 × 36, 48 × 48 × 10, and 48 × 48 × 12, for Bi${}_{2}$Te${}_{3}$, PbBi${}_{2}$Te${}_{4}$, PbBi${}_{4}$Te${}_{7}$ and Pb${}_{2}$Bi${}_{2}$Te${}_{5}$, respectively.

The surface states of Bi${}_{2}$Te${}_{3}$ and PbBi${}_{2}$Te${}_{4}$, have been calculated from 2D film structure made of six quintuple-layer slabs and six septuple-layer slabs, respectively. The optimization of the films has been performed with a vacuum height of 15 Å to avoid the artificial interaction between atom layers. The k-meshes used to sample the Brillouin zone have been set to 10 × 10 × 1. The parameters of the total energy and atomic forces’ convergence, RmtKmax and RMT values are identical to those used in the self-consistent calculations of the bulk compounds.

## 3. Results and Discussion

### 3.1. Compounds’ Structural Information

**Figure 1:**
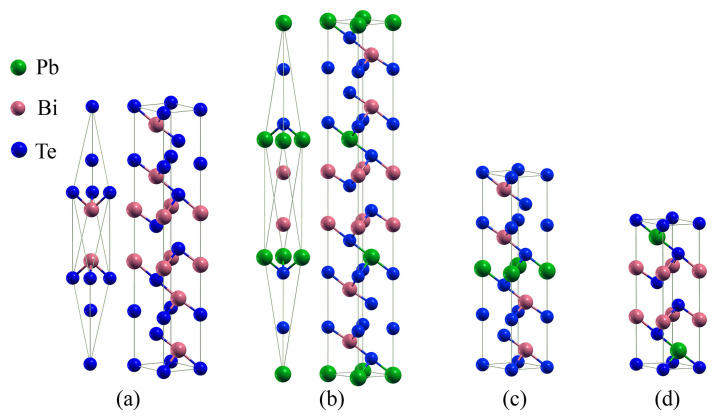
(**a**) Primitive and conventional cell of Bi${}_{2}$Te${}_{3}$, (**b**) primitive and conventional cell of PbBi${}_{2}$Te${}_{4}$, (**c**) conventional cell of PbBi${}_{4}$Te${}_{7}$, (**d**) conventional cell of Pb${}_{2}$Bi${}_{2}$Te${}_{5}$.

Bulk Bi${}_{2}$Te${}_{3}$ and PbBi${}_{2}$Te${}_{4}$ crystallize in the rhombohedral lattice system (R$\bar{3}$m space group) with 5 and 7 atoms in the primitive cell stacked along the *c*-axis, respectively. However, they can also be described with a hexagonal cell constituted by three 5-atom-layered slabs and three 7-atom-layered slabs for Bi${}_{2}$Te${}_{3}$ and PbBi${}_{2}$Te${}_{4}$, respectively (Figure 1). The PbBi${}_{4}$Te${}_{7}$ and Pb${}_{2}$Bi${}_{2}$Te${}_{5}$ compounds have a hexagonal unit cell and belong to the P$\bar{3}$m1 space group. The crystal structure of PbBi${}_{4}$Te${}_{7}$ is a 12-atom-layered one consisting of a 5-atom-layered slab and a 7-atom-layered one. Likewise, Pb${}_{2}$Bi${}_{2}$Te${}_{5}$ is constituted by one 9-atom-layered slab. Two possible atom sequences were found for Pb${}_{2}$Bi${}_{2}$Te${}_{5}$, one by Petrov [32] (–Te–Pb–Te–Bi–Te–Bi–Te–Pb–Te–) and the other one by Chatterjee [33] (–Te–Bi–Te–Pb–Te–Pb–Te–Bi–Te–). In the present study, we used the most stable sequence as found by Ma et al. [34], which corresponds to the Chatterjee one. Pb${}_{2}$Bi${}_{2}$Te${}_{5}$ with this sequence was found to be a semi-conducting compound. In all of these structures, the slabs are linked together by Te–Te interactions.

The exchange-correlation functionals we used for structure optimization were the Local Density Approximation (LDA) [35,36], the Perdew-Burke-Ernzerhof (PBE) [37] as well as the rev-vdw-DF2 [38] to account for Van der Waals forces that have to be considered in these compounds due to the weak Te–Te interactions between the slabs of the n(PbTe)-m(Bi${}_{2}$Te${}_{3}$) system. The optimized lattice parameters have been determined from total energy minimization with respect to the crystal cell volume and c/a ratio. The fitting result was obtained by using the Birch–Murnaghan equation of state:

(1) $$E\left(V\right)=E_{0}+\frac{9V_{0}B_{0}}{16}\left\{{\left[{\left(\frac{V_{0}}{V}\right)}^{\frac{2}{3}}-1\right]}^{3}B_{P}+{\left[{\left(\frac{V_{0}}{V}\right)}^{\frac{2}{3}}-1\right]}^{2}\left[6-4{\left(\frac{V_{0}}{V}\right)}^{\frac{2}{3}}\right]\right\}$$

where $V_{0}$ and *V* represent the initial and deformed volume respectively, $B_{0}$ is the bulk modulus and $B_{P}$ is the derivative of the bulk modulus with respect to pressure. Based on the optimized lattice constants, the total residual force on all atoms was relaxed to less than the aforementioned threshold. The values of the equilibrium lattice parameters and atomic positions in each of the compounds are provided in Table 1 together with experimental data from the literature.

**Figure 2:**
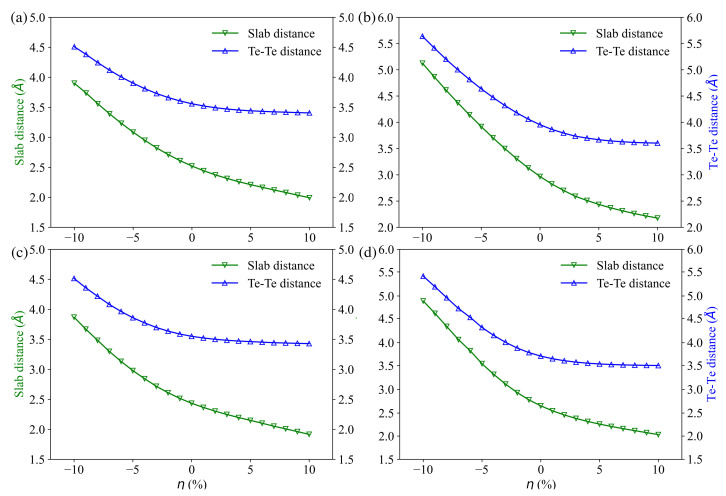
Gap distance between multi-layer slab (green lines) and Te–Te distance between adjacent slabs (blue lines) versus strain for Bi${}_{2}$Te${}_{3}$ (**a**), PbBi${}_{2}$Te${}_{4}$ (**b**), PbBi${}_{4}$Te${}_{7}$ (**c**) and Pb${}_{2}$Bi${}_{2}$Te${}_{5}$ (**d**).

Starting from the optimized structure, strains in the range −10% to 10%, which could experimentally originate from epitaxial strains in thin films [42], have been applied to *a* and *b* (b=a) lattice parameters. Based on the calculated mechanical properties [34] of the Pb${}_{2}$Bi${}_{2}$Te${}_{5}$ compound, we assume that all the compounds of interest can withstand such strains. Indeed, the bulk modulus, shear modulus and Young modulus amount to 45.6, 32.0 and 77.9 GPa respectively, for Pb${}_{2}$Bi${}_{2}$Te${}_{5}$. The strain η is calculated as $\eta =\left(a-a_{0}\right)/a_{0}$. Then, the interlayer distances have been optimized for all 4 compounds under the applied strain. Figure 2 shows the calculated inter-slab distance (denoted slab distance) and the Te–Te distance between adjacent slabs with respect to strain (compressive strain when η<0, tensile strain when η>0). Under an increasing compressive strain, slab distance and Te–Te distance between adjacent slabs increase simultaneously for all four compounds, with the latter increasing more rapidly than the former. Further, for PbBi${}_{2}$Te${}_{4}$ and Pb${}_{2}$Bi${}_{2}$Te${}_{5}$, both the Te–Te distance and the slab distance increase more rapidly than for Bi${}_{2}$Te${}_{3}$ and PbBi${}_{4}$Te${}_{7}$, indicating lower van der Waals interactions between the Te atoms. By contrast, under an increasing tensile strain, slab distance and Te–Te distance between adjacent slabs decrease simultaneously for all four compounds, but for all the compounds, the Te–Te distance decreases less than the slab distance.

**Figure 3:**
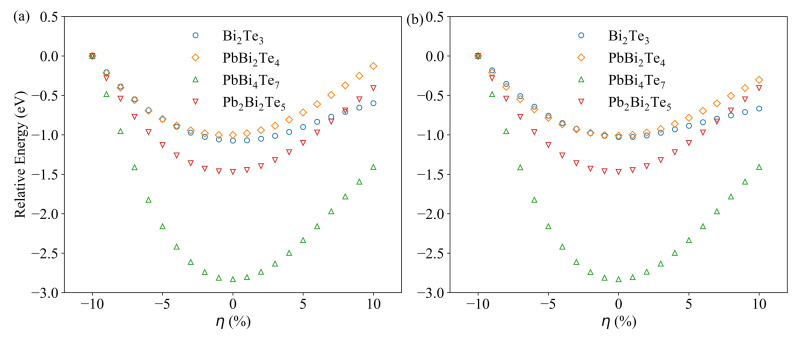
Evolution of the relative total energy as a function of strain without (**a**) and with SOC (**b**). The reference energy is taken as the energy of the structures under 10% compressive strain.

Figure 3 shows the relative total energy of the optimized structures as a function of strain calculated without spin-orbit coupling (SOC) (Figure 3a) and with SOC (Figure 3b). The reference energy is taken as the energy of the structures under 10% compressive strain, which corresponds in all cases to the highest value. The SOC effect has been investigated owing to the presence of Pb and Bi heavy metal elements in the n(PbTe)-m(Bi${}_{2}$Te${}_{3}$) compounds. One can see that for all compounds, with and without SOC, the minimum energy corresponds to a very low strain (η<2%), testifying to the stability of the unstrained structure. The SOC effect is very weak, and the biggest difference is observed for the relative energy of Bi${}_{2}$Te${}_{3}$ and PbBi${}_{2}$Te${}_{4}$ under tensile strains. By contrast, as shown in our previous study [34], the SOC effects are non-negligible on both the electronic and the thermoelectric properties. Therefore, the SOC effect has been accounted for in the forthcoming calculations.

### 3.2. Electronic Structure

**Figure 4:**
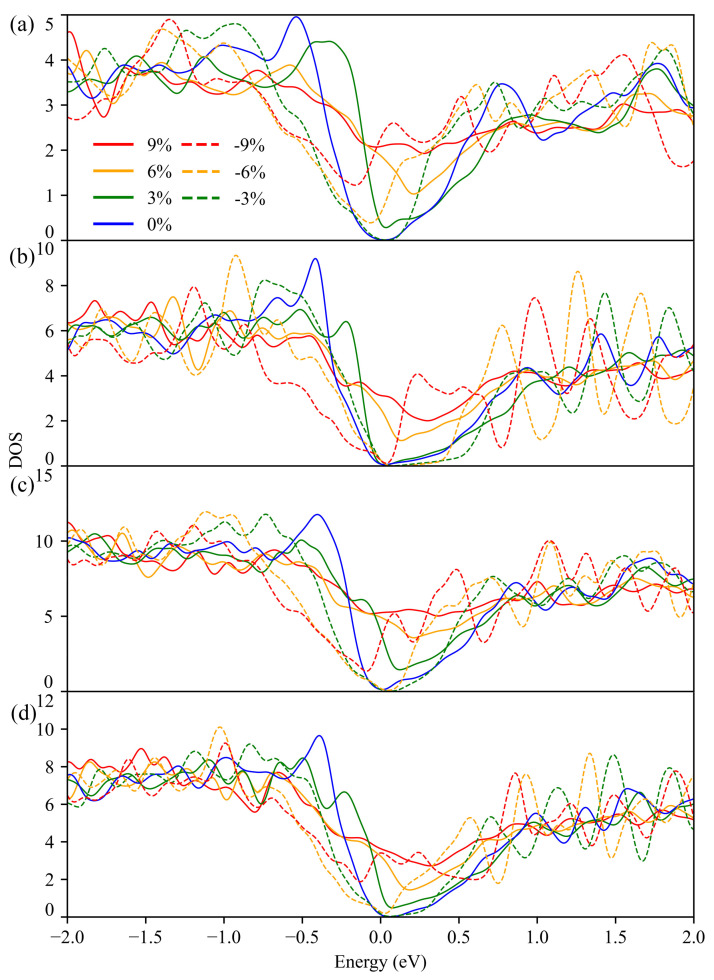
DOS of Bi${}_{2}$Te${}_{3}$ (**a**), PbBi${}_{2}$Te${}_{4}$ (**b**), PbBi${}_{4}$Te${}_{7}$ (**c**) and Pb${}_{2}$Bi${}_{2}$Te${}_{5}$ (**d**) under strains calculated with the PBE functional.

The calculated DOS of Bi${}_{2}$Te${}_{3}$, PbBi${}_{2}$Te${}_{4}$, PbBi${}_{4}$Te${}_{7}$ and Pb${}_{2}$Bi${}_{2}$Te${}_{5}$ with and without strain are depicted in Figure 4. On the basis of the conduction band minimum (CBM) and valence band maximum (VBM) positions, all the strain-free compounds are found to be indirect semiconductors. The VBM of Bi${}_{2}$Te${}_{3}$ and PbBi${}_{2}$Te${}_{4}$ locates along the L-Z path, while the CBM locates along the Z-F path (see Supplementary Figures S1 and S2), which is in agreement with previously reported data [43]. Under 3% compressive strain, irrespective of the compounds, the DOS show a semiconducting behavior. At 6%, all the compounds are semiconductors except Bi${}_{2}$Te${}_{3}$, and at 9%, they are all metals except PbBi${}_{2}$Te${}_{4}$. Under tensile strains, all the compounds are metals except PbBi${}_{2}$Te${}_{4}$ at 3%.

**Figure 5:**
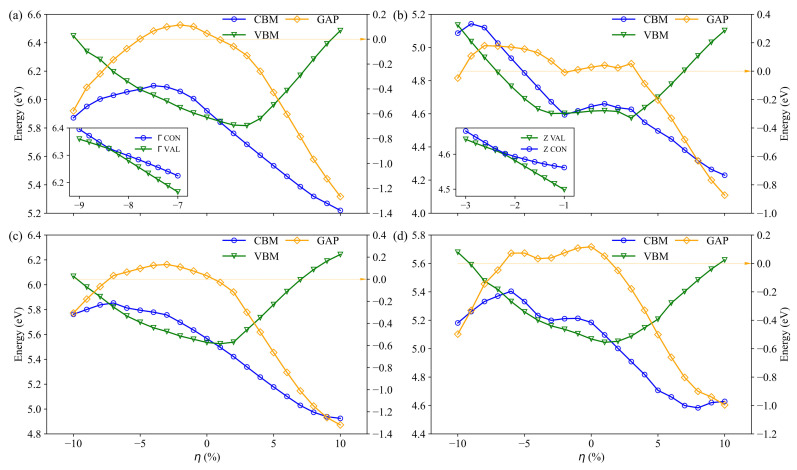
Evolution of CBM and VBM energies, and energy gap of Bi${}_{2}$Te${}_{3}$ (**a**), PbBi${}_{2}$Te${}_{4}$ (**b**), PbBi${}_{4}$Te${}_{7}$ (**c**) and Pb${}_{2}$Bi${}_{2}$Te${}_{5}$ (**d**) with respect to strain. The inset in (**a**) represents the CBM and VBM at the Γ point for Bi${}_{2}$Te${}_{3}$ and that in (**b**) represents the CBM and VBM at the Z point for PbBi${}_{2}$Te${}_{4}$.

To give a clear evolution of the semiconductor-to-metal transition, we have precisely calculated the CBM and VBM energy values from the DOS using a very dense k-point mesh. The CBM and VBM energy evolution as a function of strain is depicted in Figure 5, as well as the energy gap ($E_{\mathrm{CBM}}-E_{\mathrm{VBM}}$).

In absence of strain, Bi${}_{2}$Te${}_{3}$, PbBi${}_{2}$Te${}_{4}$, PbBi${}_{4}$Te${}_{7}$ and Pb${}_{2}$Bi${}_{2}$Te${}_{5}$ are narrow semiconductors. The compounds remain semiconductors in a wide range of strain: from −4% to 0% for Bi${}_{2}$Te${}_{3}$, from −9% to −3% and −1% to +3% for PbBi${}_{2}$Te${}_{4}$, from −7% to 0% for PbBi${}_{4}$Te${}_{7}$ and −6% to +1% for Pb${}_{2}$Bi${}_{2}$Te${}_{5}$. As it turns out, the range of non-zero band gaps is larger under compressive strains than under tensile ones. Insets in Figure 5a, b show the evolution of VBM and CBM energy versus strains at Z point for PbBi${}_{2}$Te${}_{4}$ and at Γ point for Bi${}_{2}$Te${}_{3}$. The energy gap of PbBi${}_{2}$Te${}_{4}$ with 3% compressive strain is very small and direct at Z point, whereas it is indirect for most of the strained compounds. The energy difference at the Γ point for Bi${}_{2}$Te${}_{3}$ vanishes for a compressive strain of about 8%.

### 3.3. Transport Properties

**Figure 6:**
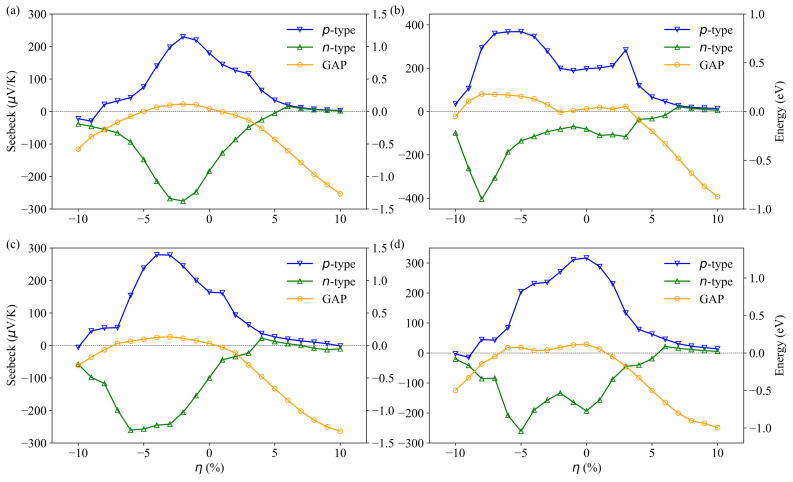
Maximum Seebeck coefficient and band-gap energy of Bi${}_{2}$Te${}_{3}$ (**a**), PbBi${}_{2}$Te${}_{4}$ (**b**), PbBi${}_{4}$Te${}_{7}$ (**c**) and Pb${}_{2}$Bi${}_{2}$Te${}_{5}$ (**d**) vs. strain at 300 K. The blue and green lines correspond to the Seebeck coefficient for *p*-type and *n*-type doping, respectively. The charge carrier concentrations range from $10^{17}$ to $10^{22}$ cm${}^{-3}$. The orange lines correspond to the band-gap energy.

Based on the calculated electronic structure, the Seebeck coefficient (*S*) of Bi${}_{2}$Te${}_{3}$, PbBi${}_{2}$Te${}_{4}$, PbBi${}_{4}$Te${}_{7}$ and Pb${}_{2}$Bi${}_{2}$Te${}_{5}$ has been investigated. Figure 6 shows the calculated *S* at 300 K as a function of doping level under different strains for all the compounds. We found that the maximum Seebeck coefficients for *p*-type Bi${}_{2}$Te${}_{3}$, PbBi${}_{2}$Te${}_{4}$, PbBi${}_{4}$Te${}_{7}$ and Pb${}_{2}$Bi${}_{2}$Te${}_{5}$ are located at −2%, −6%, −3% and 0% strain, which are near the maximum band gaps of each compound. For Bi${}_{2}$Te${}_{3}$, with increasing compressive strain up to −2%, the Seebeck coefficient increases for both *p*-type and *n*-type doping. For higher compressive strains, the Seebeck coefficient decreases while remaining higher than that of the strain-free compound up to −3% (*p*-type) and −4% (*n*-type). The maximum Seebeck coefficient for PbBi${}_{2}$Te${}_{4}$ is obtained at a compressive strain of −6% for *p*-type doping and −8% for *n*-type doping. In order to explain the difference in the Seebeck coefficients under strain, the tight relationship between *S* and electronic structure has been studied. Following the band theory, the Seebeck coefficient can be described as [44,45]:

(2) $$S=-\frac{k_{B}^{2}}{e}\frac{1}{n\mu_{n}+p\mu_{p}}\left\{\left(2-\frac{E_{F}}{kT}\right)n\mu_{n}-\left(2-\frac{E_{F}+E_{G}}{kT}\right)p\mu_{p}\right\}$$

(3) $$S=-\frac{k_{B}}{e}\frac{1}{n\mu_{n}+p\mu_{p}}\left\{\left[2+\ln\left(\frac{N_{c}}{n}\right)\right]n\mu_{n}-\left[2+\ln\left(\frac{N_{v}}{p}\right)\right]p\mu_{p}\right\}$$

where $k_{B}$ is the Boltzmann constant, *e* is the elementary charge, $E_{F}$ and $E_{G}$ are the Fermi energy and the band-gap energy, $N_{v}$ and $N_{c}$ are the effective DOS of the valence and conduction bands, $\mu_{n}$ and $\mu_{p}$ are the mobility of electrons and holes and *n* and *p* are the number of electrons and holes, respectively.

The Seebeck coefficient depends on the carrier concentration, the effective DOS of the conduction and valence band, band-gap width and Fermi energy. By means of strain-modified DOS, the Seebeck coefficient can be changed accordingly. The maximum Seebeck coefficients at 300 K of PbBi${}_{2}$Te${}_{4}$ without strain are 198 μV/K and −81 μV/K for *p*-type and *n*-type doping, respectively. These results are in agreement with the effective DOS values and the band gap, i.e., the value of the valence band is higher than that of the conduction one (Figure 4b). Besides, due to the similar effective DOS of the valence band and conduction band of Bi${}_{2}$Te${}_{3}$, the Seebeck coefficient magnitudes for *p*-type and *n*-type doping are very close. As can be seen in Figure 4d and Supplementary Figure S2, in the PbBi${}_{2}$Te${}_{4}$ compound under −9% compressive strain, many conduction band valleys with energy difference less than 0.3 eV show up around the Fermi level, leading to a high effective DOS and hence to a high Seebeck coefficient for *n*-type doping. Similarly, several valence band valleys show up around the Fermi level in the same compound under −6% compressive strains, which is in agreement with the high Seebeck coefficient calculated for *p*-type doping.

**Figure 7:**
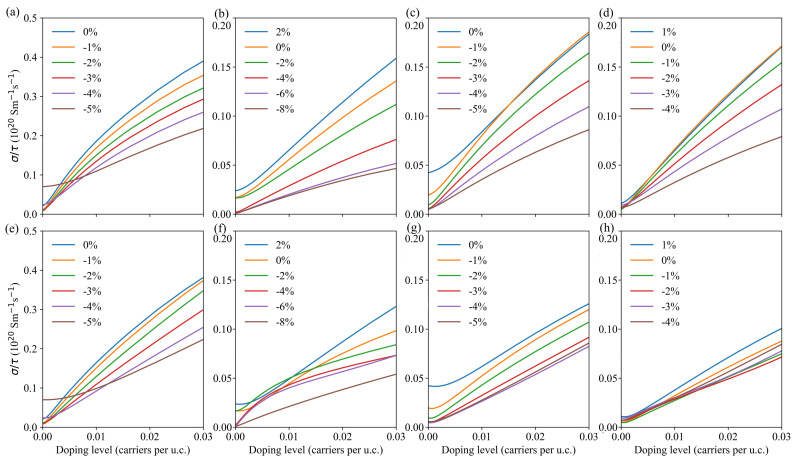
τ-scaled electronic conductivity of Bi${}_{2}$Te${}_{3}$ (**a**,**e**), PbBi${}_{2}$Te${}_{4}$ (**b**,**f**), PbBi${}_{4}$Te${}_{7}$ (**c**,**g**) and Pb${}_{2}$Bi${}_{2}$Te${}_{5}$ (**d**,**h**) vs. carrier concentrations for various compressive and tensile strains. Top figures correspond to *p*-type doping and bottom figures to *n*-type doping.

For solving the Boltzmann transport equation, the BoltzTraP2 code uses the relaxation time approximation, and therefore the scattering time τ remains undefined. In general, for a given structure, τ depends on temperature and doping level. We can obtain a temperature-dependent τ from the so-called deformation potential theory based on the deformation potential, the carriers’ effective mass and the elastic constants. Nevertheless, with the changes of strain, the effective mass of carriers also changes, which would make it computationally difficult to evaluate the scattering time for each and every case. Therefore, we have kept τ as is in the electronic conductivity σ. Due to the low Seebeck coefficient in metallic compounds, only the electronic conductivity results for strained compounds with opened band gap are reported in the following. The corresponding strain ranges are −5% to 0% for Bi${}_{2}$Te${}_{3}$ and PbBi${}_{4}$Te${}_{7}$, −8% to 2% for PbBi${}_{2}$Te${}_{4}$ and −4% to 1% for Pb${}_{2}$Bi${}_{2}$Te${}_{5}$. Figure 7 shows the calculated σ/τ at 300 K as a function of doping level for various strains. In most cases, the electrical conductivity decreases with the increase of compressive strain for both *p*-type and *n*-type doping, at least for high doping levels. The peculiar behavior of Pb${}_{2}$Bi${}_{2}$Te${}_{5}$ can be noticed for *n*-type doping as the electrical conductivity is rather insensitive to the applied strains. Except for Bi${}_{2}$Te${}_{3}$, which shows similar evolutions of the electrical conductivity under strains for both *p*- and *n*-type doping, the electrical conductivity is more sensitive to the applied strain for *p*-type doping than for *n*-type doping. This is probably due to the larger dispersion of the conduction bands of PbBi${}_{2}$Te${}_{4}$, PbBi${}_{4}$Te${}_{7}$ and Pb${}_{2}$Bi${}_{2}$Te${}_{5}$ than that of the valence bands around the Fermi level (see Figure 4) in the range of strains with opened gaps.

**Figure 8:**
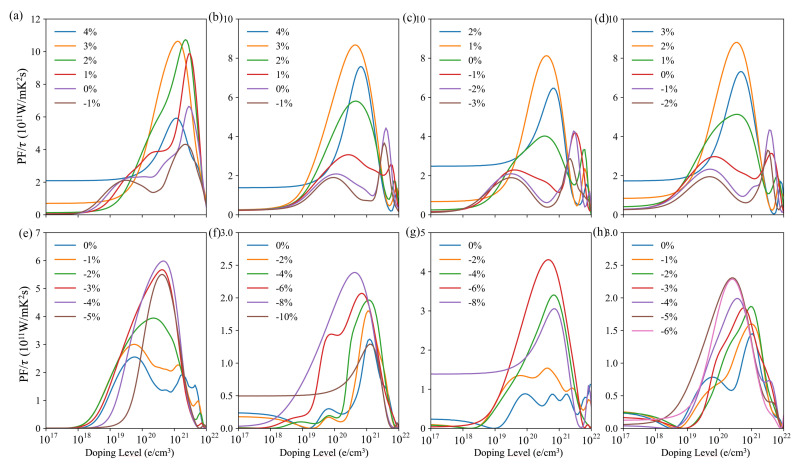
τ-scaled power factor of Bi${}_{2}$Te${}_{3}$ (**a**,**e**), PbBi${}_{2}$Te${}_{4}$ (**b**,**f**), PbBi${}_{4}$Te${}_{7}$ (**c**,**g**) and Pb${}_{2}$Bi${}_{2}$Te${}_{5}$ (**d**,**h**) vs. carrier concentrations for various compressive and tensile strains. Top figures correspond to *p*-type doping and bottom figures to *n*-type doping.

According to the transport equations, *S* and σ evolve in opposite ways from one another. This is still valid for our results under strains, which indicates that strains could not de-correlate these quantities. Hence, the power factor, PF = $S^{2}\sigma$, is essential to evidence the evolution of the material efficiency with respect to the applied strains. The PF of the investigated *p*-type and *n*-type compounds is depicted in Figure 8 for various compressive and tensile strains that have been chosen in order to better evidence the evolution of the power factor. Overall, the power factor is improved by applying strains. Specifically, the power factor for *p*-type compounds tends to increase upon tensile strains, whereas that of the n-type compounds tends to increase under compressive strains. The largest PF values are obtained for the *p*-type compounds under tensile strains.

The calculated power factors are fairly good if one considers τ to amount to a commonly used value of $10^{-14}$ s [46,47,48]. Some of these values are, however, calculated for closed energy gaps, which highlights the difficulty to optimize the thermoelectric properties of these materials.

### 3.4. Charge and Bonding Evolution under Strains

**Figure 9:**
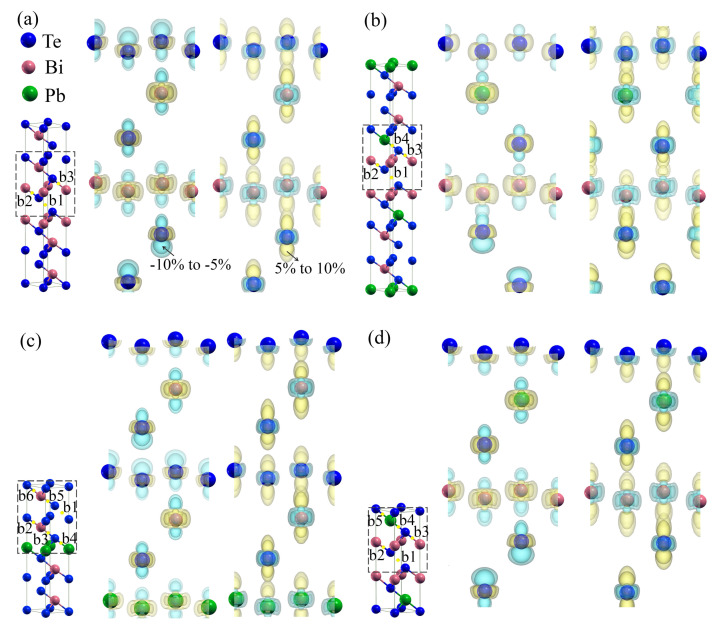
Charge density differences in Bi${}_{2}$Te${}_{3}$ (**a**), PbBi${}_{2}$Te${}_{4}$ (**b**), PbBi${}_{4}$Te${}_{7}$ (**c**) and Pb${}_{2}$Bi${}_{2}$Te${}_{5}$ (**d**) under compressive strains (−10% and −5%) and tensile strains (5% and 10%). The isosurface is set to 0.05 e/bohr${}^{3}$ for both accumulation (yellow) and depletion (cyan). The arrows indicate the evolution of the charge density difference as the strain evolves. b1–b6: Bond critical points.

In order to complete the investigation, we have calculated charge density differences between strained and unstrained structures ($\rho_{\mathrm{diff}}=\rho_{\mathrm{unstrained}}-\rho_{\mathrm{strained}}$). Table 1 shows a great difference in lattice parameters and atomic positions between the strained and the unstrained structures. In order to compare the difference of charge density between strained and unstrained structures, we have meshed the cells in the [001] direction with grids leading to the same number of ac-planes (00x) between each pair of adjacent atoms. Figure 9 displays the charge differences in Bi${}_{2}$Te${}_{3}$, PbBi${}_{2}$Te${}_{4}$, PbBi${}_{4}$Te${}_{7}$ and Pb${}_{2}$Bi${}_{2}$Te${}_{5}$ for compressive (−10% and −5%) and tensile strains (5% and 10%) with an iso-surface of 0.05 e/bohr${}^{3}$. The yellow color represents charge increase, while the cyan one corresponds to charge decrease. A compressive strain leads to charge increase in the in-plane (ab-axis) direction, and decrease in the cross-plane (*c*-axis) direction, with the decrease being larger for a higher value of the compressive strain (cf. arrow in Figure 9). Conversely, opposite results are obtained under tensile strain, which reveals that the charge density between the slabs increases when going from a compressive strain to a tensile one. As a consequence, the van der Waals bonds located between the slabs should be strengthened under a tensile strain.

**Figure 10:**
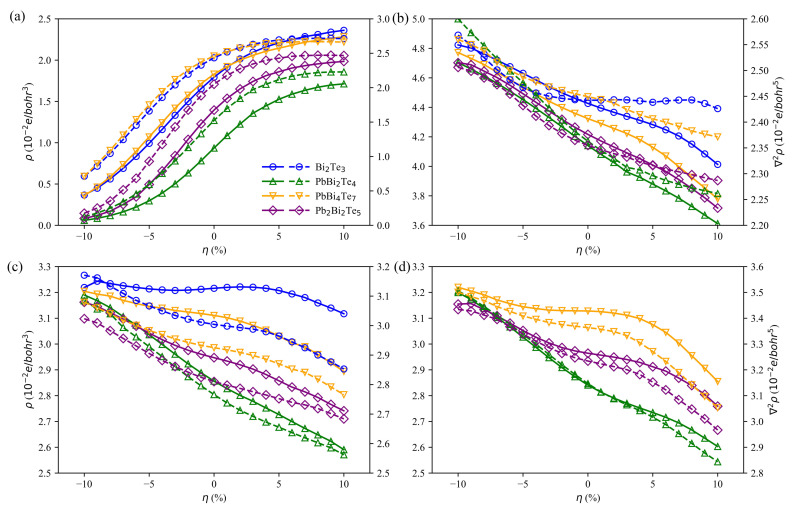
Charge density, ρ (solid lines), and charge density Laplacian, $\nabla^{2}\rho$ (dashed lines), at various bond critical points (b1 in (**a**), b2 in (**b**), b3 in (**c**) and b4 in (**d**)) for Bi${}_{2}$Te${}_{3}$, PbBi${}_{2}$Te${}_{4}$, PbBi${}_{4}$Te${}_{7}$ and Pb${}_{2}$Bi${}_{2}$Te${}_{5}$ w.r.t. strain. The positions of b1, b2, b3 and b4 are defined in Figure 9.

To further understand the evolution of the bonds under strains, we have used the quantum theory of atoms in molecules (QTAIM) [49]. All irreducible bond critical points (BCP) in Bi${}_{2}$Te${}_{3}$ and PbBi${}_{2}$Te${}_{4}$ are considered. Due to similar properties between b2 and b5, and b3 and b6 in PbBi${}_{4}$Te${}_{7}$ (see Supplementary Figure S5), and b4 and b5 in Pb${}_{2}$Bi${}_{2}$Te${}_{5}$ (see Supplementary Figure S6), only b1 to b4 BCPs are taken into account for PbBi${}_{4}$Te${}_{7}$ and Pb${}_{2}$Bi${}_{2}$Te${}_{5}$. The charge density, ρ, and its Laplacian, $\nabla^{2}\rho$, at these BCPs, presented in Figure 10, reveal that the strain affects the bonds between slabs much more than the in-slab ones. As shown by the positive values of the electron density Laplacian irrespective of the strain, there is no charge accumulation at the considered BCPs. The values of the Laplacian, $\nabla^{2}\rho$, also called valence shell charge concentrations (VSCC), and the charge density, ρ, at the in-slab BCPs diminish when going from a compressive strain to a tensile one, indicating that the in-slab bonds become weaker, while the interaction between slabs is getting stronger. These results are in agreement with those related to the charge density differences presented above.

**Figure 11:**
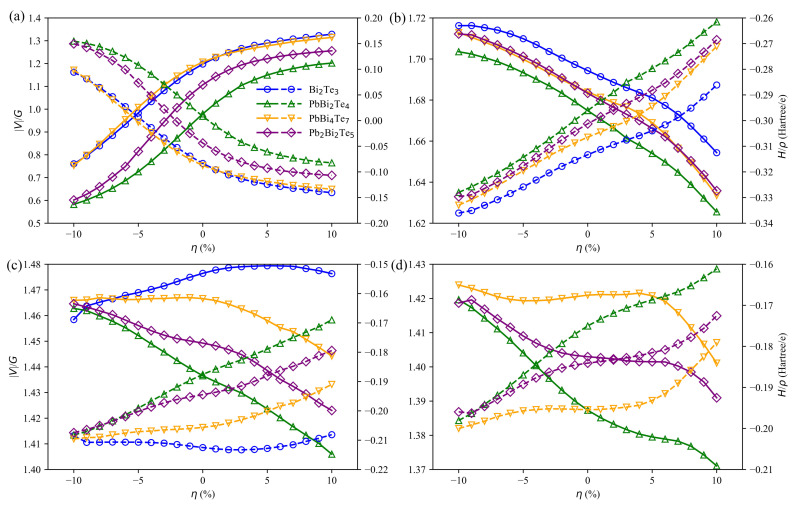
|V|/G (solid lines) and H/ρ (dashed lines) at various bond critical points (b1 in (**a**), b2 in (**b**), b3 in (**c**) and b4 in (**d**)) for Bi${}_{2}$Te${}_{3}$, PbBi${}_{2}$Te${}_{4}$, PbBi${}_{4}$Te${}_{7}$ and Pb${}_{2}$Bi${}_{2}$Te${}_{5}$ w.r.t. strain. The positions of b1, b2, b3 and b4 are defined in Figure 9.

Based on Bader’s theory of Atoms in Molecules, the sign of $\nabla^{2}\rho$ can be used to differentiate the closed-shell (CS) interactions from the shared-shell (SS) interactions. Closed-shell interactions (ionic, H-bonds and vdW) have a large positive value of $\nabla^{2}\rho$, |V|/G<1 and a small ρ. Conversely, $\nabla^{2}\rho <0$, |V|/G>2 and a large ρ are expected for shared interactions (covalent or polar bonds). In our study, all BCPs, except the Te-Te ones, show a small value of ρ, positive $\nabla^{2}\rho$ with 1<|V|/G<2 (Figure 11). As a consequence, the corresponding bonds cannot be considered as pure covalent nor pure closed-shell ones. They belong to a transit region identified by Espinosa [50] and confirmed by Dinda [51]. By contrast, as expected, the Te-Te bonds with a small value of ρ, positive $\nabla^{2}\rho$ with |V|/G<1 correspond to pure closed-shell interactions. When going from compressive to tensile strain, the bond degree defined by Espinosa as BD=H/ρ goes from a positive value to a negative one, leading, as already mentioned above, to a bond strengthening for the van der Waals bonds located between the slabs.

### 3.5. Topological Properties of Bi${}_{2}$Te${}_{3}$ and PbBi${}_{2}$Te${}_{4}$ under Peculiar Strains

Bi${}_{2}$Te${}_{3}$ and PbBi${}_{2}$Te${}_{4}$ have been already identified as 3D topological insulators (TI) [52,53,54]. As discussed above, band structure changes along with the strain. In addition, several Dirac-like cones have been observed along the high symmetry path. Based on the results shown in Figure 5a,b, two interesting strained electronic structures, presenting gapless bands and Dirac-like dispersion, have been selected to investigate the topological properties of Bi${}_{2}$Te${}_{3}$ and PbBi${}_{2}$Te${}_{4}$. The corresponding strain values are −8.4% and −2.2% for Bi${}_{2}$Te${}_{3}$ and PbBi${}_{2}$Te${}_{4}$, respectively. All the results presented hereafter have been obtained with these strains.

**Figure 12:**
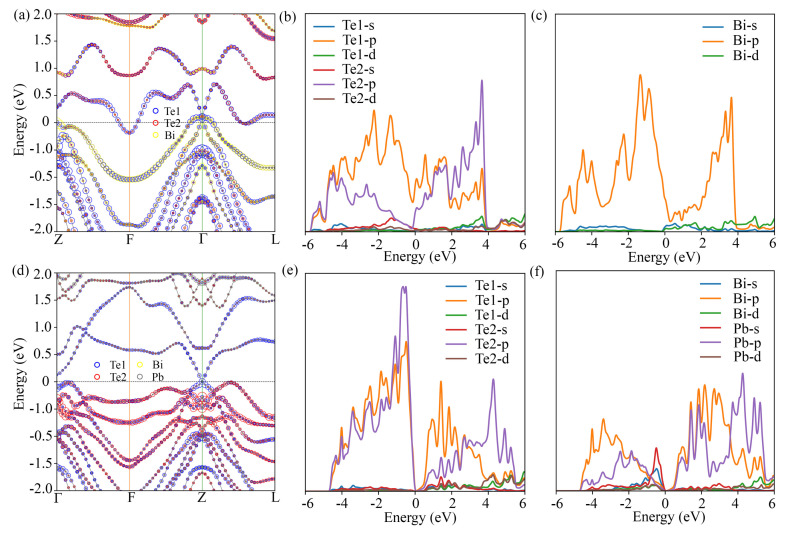
Band structure and PDOS of bulk Bi${}_{2}$Te${}_{3}$ (**a**–**c**) and bulk PbBi${}_{2}$Te${}_{4}$ (**d**–**f**) with SOC. The size of the colored circles is proportional to the partial weights of the atomic states.

The band structures and partial density of states (PDOS) are depicted in Figure 12. The electronic gaps are closed at the Γ and Z points for Bi${}_{2}$Te${}_{3}$ and PbBi${}_{2}$Te${}_{4}$, respectively. The PDOS indicates that the main contributing orbitals close to the Fermi level are Te 5p, Bi 6p and Bi 6s for both Bi${}_{2}$Te${}_{3}$ and PbBi${}_{2}$Te${}_{4}$. For PbBi${}_{2}$Te${}_{4}$, Pb 6s orbitals also significantly contribute. More specifically, the valence band of Bi${}_{2}$Te${}_{3}$ near Γ is composed predominantly of Te states, whereas the conduction band is contributed by Bi states. A similar situation is found at Z point for PbBi${}_{2}$Te${}_{4}$. Such an inversion of gap edges may indicate a change of the parity of the occupied states, revealing a promising topological insulator (TI) [55].

**Figure 13:**
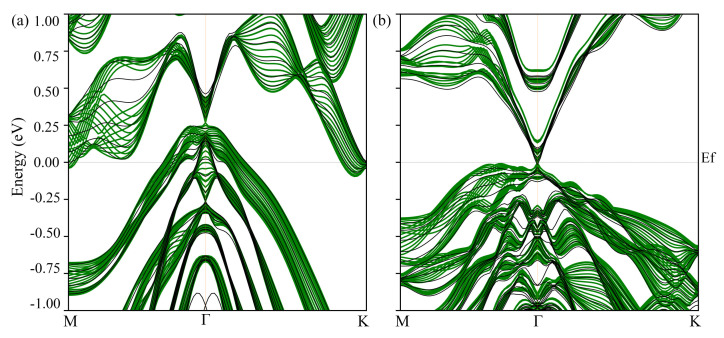
Surface states (black lines) and projected band structure on the (001) plane (green lines) of Bi${}_{2}$Te${}_{3}$ (**a**) and PbBi${}_{2}$Te${}_{4}$ (**b**).

In order to investigate the surfaces’ electronic states, thin film structures of Bi${}_{2}$Te${}_{3}$ and PbBi${}_{2}$Te${}_{4}$, both with a P$\bar{3}$m1 symmetry, have been designed. These structures, which consist of six 5-atom-layered slabs (5L–5L–5L–5L–5L–5L) and six 7-atom-layered slabs (7L–7L–7L–7L–7L–7L), leading to 30 atomic layers and 42 atomic layers respectively, have been investigated under strain. The calculated band structures are shown in Figure 13. As mentioned above, TIs exhibit gapless bands and Dirac-like dispersion, however a Dirac-like cone is only observed in PbBi${}_{2}$Te${}_{4}$ (Figure 13b). The Dirac-like cone observed in bulk Bi${}_{2}$Te${}_{3}$ disappears in the Bi${}_{2}$Te${}_{3}$ thin film (Figure 13a).

The time-reversal invariant energy bands in 3D TI are characterized by 4 $Z_{2}$ invariants distinguishing 16 phases with 2 general classes: weak (WTI) and strong (STI) topological insulators [56,57]. Due to the spatial inversion symmetry of hexagonal Bi${}_{2}$Te${}_{3}$ and PbBi${}_{2}$Te${}_{4}$, the determination of $Z_{2}$ invariants is simplified. Indeed, they can be obtained from the parity of the occupied states at the eight time-reversal invariant momenta $\Gamma_{i}$ in the Brillouin zone using the following formula [58]:

(4) $${\left(-1\right)}^{v_{0}}=\prod_{i=1}^{8}\delta_{i}$$

where $\delta_{i}=\prod_{m=1}^{N}\xi_{2m}\left(\Gamma_{i}\right)$ with *N* are the Kramers doublets in the valence band, and $\xi_{2m}\left(\Gamma_{i}\right)$ is the parity of the 2m${}^{\mathrm{th}}$ occupied state at $\left(\Gamma_{i}\right)$.

In the 3D Brillouin zone, the eight time-reversal invariant momenta (TRIM), namely Γ(0, 0, 0), M(0, 0, 0.5), (0, 0.5, 0), (0.5, 0, 0), L(0.5, 0, 0.5), (0, 0.5, 0.5), (0.5, 0.5, 0) and A(0.5, 0.5, 0.5), yield the four independent $Z_{2}$ indices, $v_{0}$ ($v_{1}v_{2}v_{3}$). Strong TIs have a non-zero $v_{0}$, whereas weak ones are characterized by $v_{0}=0$ and $v_{1},v_{2}$ or $v_{3}\neq 0$. In our study, the $\delta_{i}$ have been calculated by accounting for 54 and 72 isolated valence bands near the Fermi level for Bi${}_{2}$Te${}_{3}$ and PbBi${}_{2}$Te${}_{4}$, respectively. Table 2 presents the calculated values of the $\delta_{i}$ and $Z_{2}$ invariants of strained Bi${}_{2}$Te${}_{3}$ and PbBi${}_{2}$Te${}_{4}$. The strained Bi${}_{2}$Te${}_{3}$ is found to be topologically trivial. Therefore, the Dirac-like band (Figure 12a) can be considered as an accidental degeneracy. On the other hand, based on calculated $Z_{2}$, the strained PbBi${}_{2}$Te${}_{4}$ can be estimated as a weak TI.

## 4. Conclusions

We have investigated the effect of various tensile and compressive strains on the electronic structure and transport properties of Bi${}_{2}$Te${}_{3}$, PbBi${}_{2}$Te${}_{4}$, PbBi${}_{4}$Te${}_{7}$ and Pb${}_{2}$Bi${}_{2}$Te${}_{5}$ by means of first-principle calculations. Our calculations reveal that Bi${}_{2}$Te${}_{3}$, PbBi${}_{2}$Te${}_{4}$, PbBi${}_{4}$Te${}_{7}$ and Pb${}_{2}$Bi${}_{2}$Te${}_{5}$ without strains are semiconductors. When applying strains, whether being tensile or compressive, a semiconductor-to-metal transition occurs for all the compounds, with the strain values corresponding to the transition being different for each compound. The range of non-zero band gaps is larger under compressive strains than under tensile ones. Seebeck coefficients’ maxima were located at −2%, −6%, −3% and 0% strain for *p*-type Bi${}_{2}$Te${}_{3}$, PbBi${}_{2}$Te${}_{4}$, PbBi${}_{4}$Te${}_{7}$ and Pb${}_{2}$Bi${}_{2}$Te${}_{5}$, respectively. These strain values correspond roughly to those related to the band-gap maxima for each compound. Besides, compressive strains induce larger Seebeck coefficients than tensile ones. Within the range of open-gap, the electrical conductivity decreases as the compressive strain increases. Electrical conductivity is more sensitive to the applied strain for *p*-type doping than for *n*-type doping. Based on the analysis of charge density differences and the quantum theory of atoms in molecules, we have found that for the van der Waals interactions located between the slabs, the charge density increases and the bond degree goes from a positive value to a negative one when going from a compressive strain to a tensile one.

Furthermore, the topological properties of Bi${}_{2}$Te${}_{3}$ (under −8.4% strain) and PbBi${}_{2}$Te${}_{4}$ (under −2.2% strain) have been investigated. The strained Bi${}_{2}$Te${}_{3}$ was found to be topologically trivial, whereas the strained PbBi${}_{2}$Te${}_{4}$ can be estimated as a weak TI.

## Figures and Tables

**Table 1:** Calculated lattice parameters (Å) and atom fractional coordinates along the c axis for Bi${}_{2}$Te${}_{3}$, PbBi${}_{2}$Te${}_{4}$, PbBi${}_{4}$Te${}_{7}$ and Pb${}_{2}$Bi${}_{2}$Te${}_{5}$ compared with the experimental ones.

		LDA	PBE	Rev-vdW-DF2	Exp.
PbTe	*a*	6.38	6.57	-	6.46 [39]
Bi${}_{2}$Te${}_{3}$	*a*	4.36	4.46	4.35	4.38 [40]
	*c*	30.00	30.75	30.21	30.49 [40]
	Te1	0	0	0	0 [40]
	Te2	0.2029	0.2116	0.2086	0.212 [40]
	Bi	0.3997	0.3991	0.4003	0.4 [40]
PbBi${}_{2}$Te${}_{4}$	*a*	4.45	4.55	4.52	4.34 [41]
	*c*	41.87	43.21	42.82	41.77 [41]
	Pb	0	0	0	
	Te1	0.1336	0.1304	01322	
	Te2	0.2873	0.2881	0.2876	
	Bi	0.4271	0.4252	0.4263	
PbBi${}_{4}$Te${}_{7}$	*a*	4.39	4.50	4.47	4.42 [7]
	*c*	22.88	23.30	23.24	24.04 [7]
	Te1	0	0	0	
	Bi1	0.0849	0.0842	0.0846	
	Te2	0.1582	0.1561	0.1568	
	Te3	0.2602	0.2624	0.2616	
	Bi2	0.3331	0.3337	0.3334	
	Te4	0.4183	0.4182	0.4183	
	Pb	0.5	0.5	0.5	
Pb${}_{2}$Bi${}_{2}$Te${}_{5}$	*a*	4.43	4.55	4.50	4.42 [33]
	*c*	17.32	17.41	17.68	17.86 [33]
	Te1	0	0	0	
	Pb	0.1099	0.1097	0.1100	
	Te2	0.2192	0.2194	0.2189	
	Bi	0.3326	0.3349	0.3323	
	Te3	0.4299	0.4294	0.4261	

**Table 2:** $\delta_{i}$ values at eight time-reversal invariant momenta and $Z_{2}$ invariant values of Bi${}_{2}$Te${}_{3}$ under −8.4% strain and PbBi${}_{2}$Te${}_{4}$ under −2.2% strain.

	$\delta_{1}$	$\delta_{2}$	$\delta_{3}$	$\delta_{4}$	$\delta_{5}$	$\delta_{6}$	$\delta_{7}$	$\delta_{8}$	$Z_{2}$
Bi${}_{2}$Te${}_{3}$	−1	−1	−1	−1	−1	−1	−1	−1	0 (0,0,0)
PbBi${}_{2}$Te${}_{4}$	−1	−1	1	1	1	1	1	1	0 (0,0.5,0)

## Data Availability

See supplementary data on MDPI website.

## Supplementary Materials

The following are available online at https://www.mdpi.com/article/10.3390/ma14154086/s1, Figure S1: Calculated bands’ structures of Bi${}_{2}$Te${}_{3}$ with PBE functional under strains, Figure S2: Calculated bands’ structures of PbBi${}_{2}$Te${}_{4}$ with PBE functional under strains, Figure S3: Calculated bands’ structures of PbBi${}_{4}$Te${}_{7}$ with PBE functional under strains, Figure S4: Calculated bands’ structures of Pb${}_{2}$Bi${}_{2}$Te${}_{5}$ with PBE functional under strains, Figure S5: |V|/G (solid lines) and ρ (dashed lines) at various critical points (b2, b3, b4 and b5) for PbBi${}_{4}$Te${}_{7}$ w.r.t strain. The positions of b2, b3, b4 and b5 are defined in Figure 9c. Figure S6: |V|/G (solid lines) and ρ (dashed lines) at various critical points (b4 and b5) for Pb${}_{2}$Bi${}_{2}$Te${}_{5}$ w.r.t strain. The positions of b4 and b5 are defined in Figure 9d.

## Abbreviations

The following abbreviations were used in this manuscript:

TE	Thermoelectric
DFT	Density functional theory
QTAIM	Quantum theory of atoms in molecules
TEG	Thermoelectric generators
PBE	Perdew-Burke-Ernzerhof
DOS	Density of states
RMT	Radius of Muffin Tin
SOC	Spin-orbit coupling
CBM	Conduction band minimum
VBM	Valence band maximum
