# Supplementary material for: Strain Effects on the Electronic and Thermoelectric Properties of n(PbTe)-m(Bi2Te3) System Compounds
Source: Materials (Basel). 2021 Jul 22;14(15):4086. doi: 10.3390/ma14154086 (PMC8348818; doi:10.3390/ma14154086)
Supplement: Supplementary file 1 [file materials-14-04086-s001.zip › supplementary.pdf]

# Supplementary Materials: Strain effects on the electronic and thermoelectric properties of n(PbTe)-m($Bi_2Te_3$) system compounds

**Weiliang Ma [1,2,†], Marie-Christine Record [1,‡,*], Jing Tian [2,†] and Pascal Boulet [2,†]**

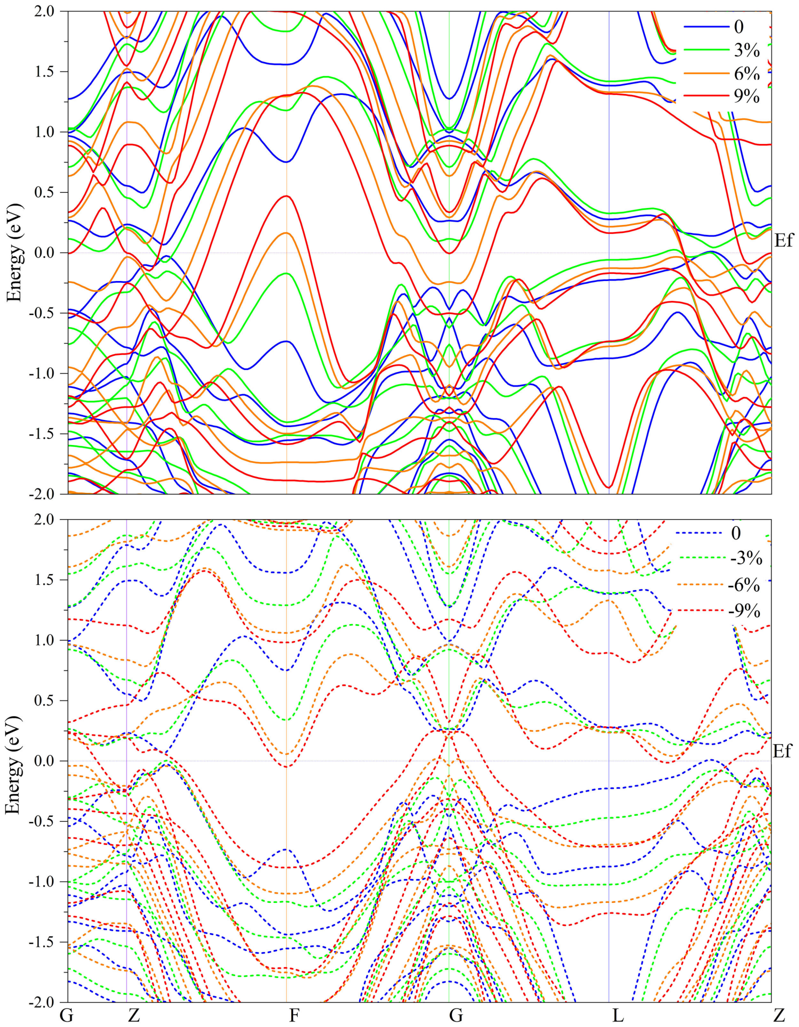


**Figure S1.** Calculated bands structures of $Bi_2Te_3$ with PBE functional under strains.

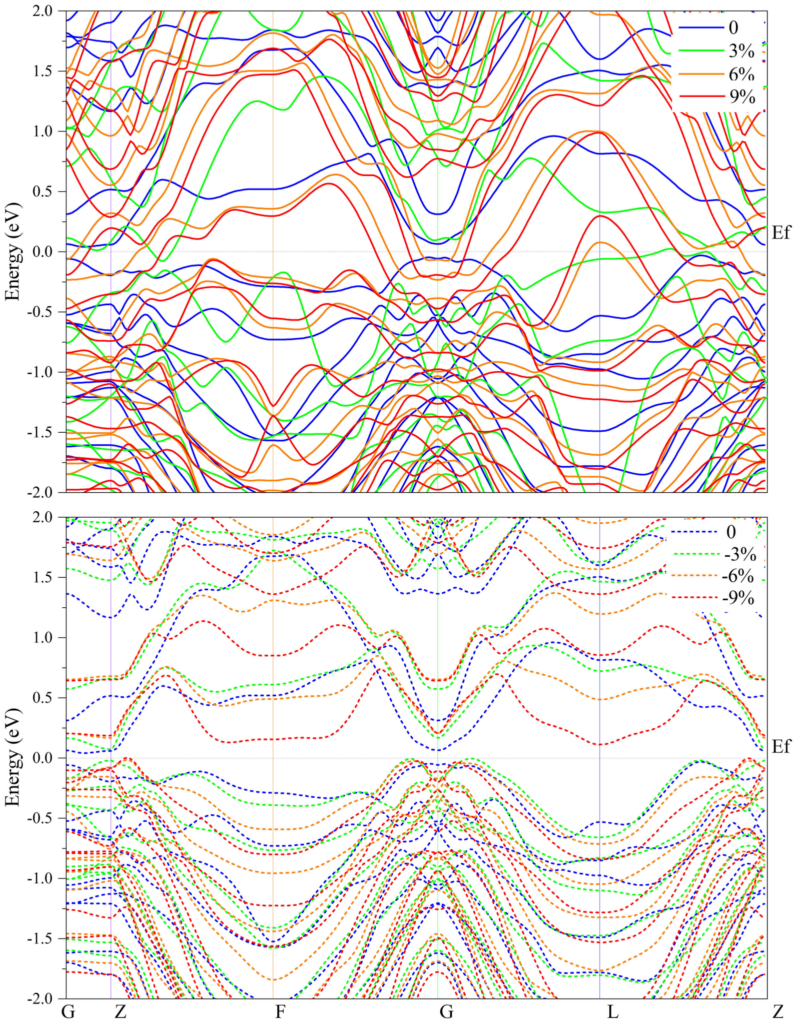


**Figure S2.** Calculated bands structures of $PbBi_2Te_4$ with PBE functional under strains.

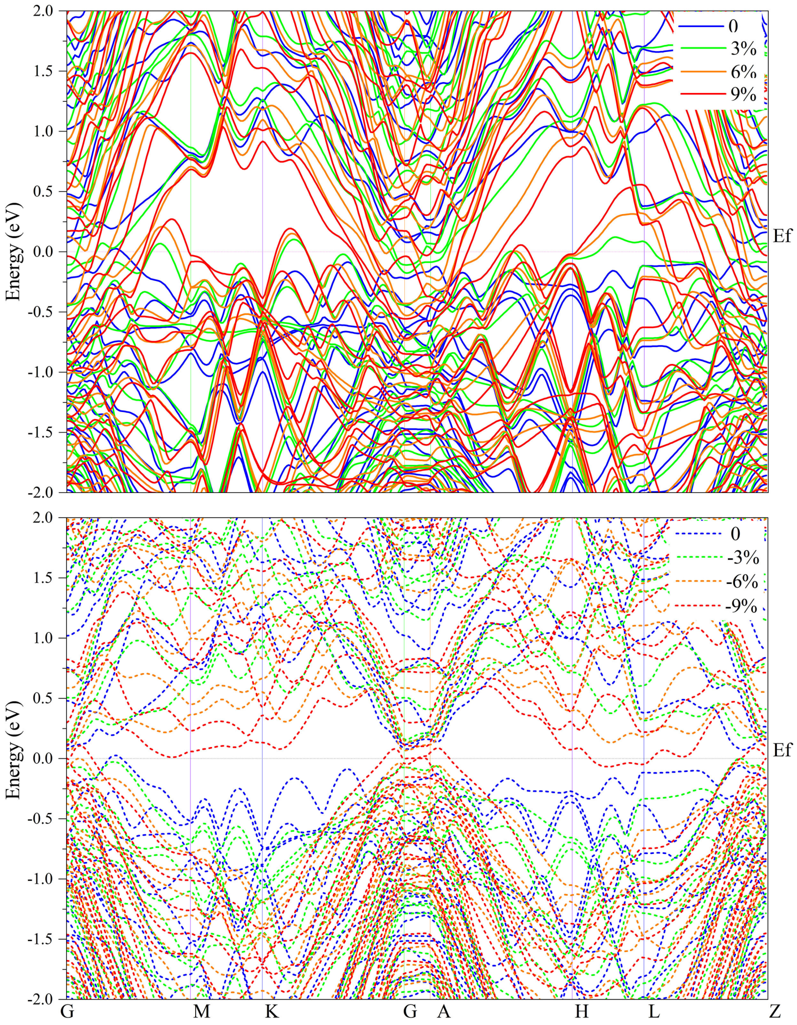


**Figure S3.** Calculated bands structures of $PbBi_4Te_7$ with PBE functional under strains.

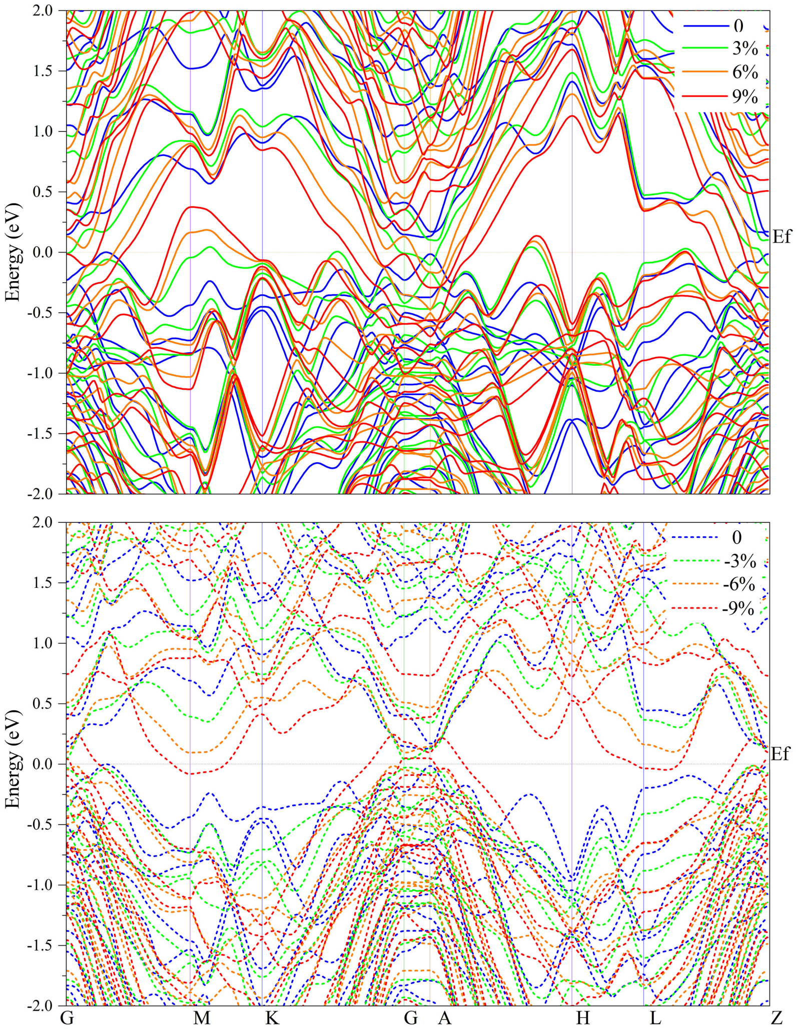


**Figure S4.** Calculated bands structures of $Pb_2Bi_2Te_5$ with PBE functional under strains.

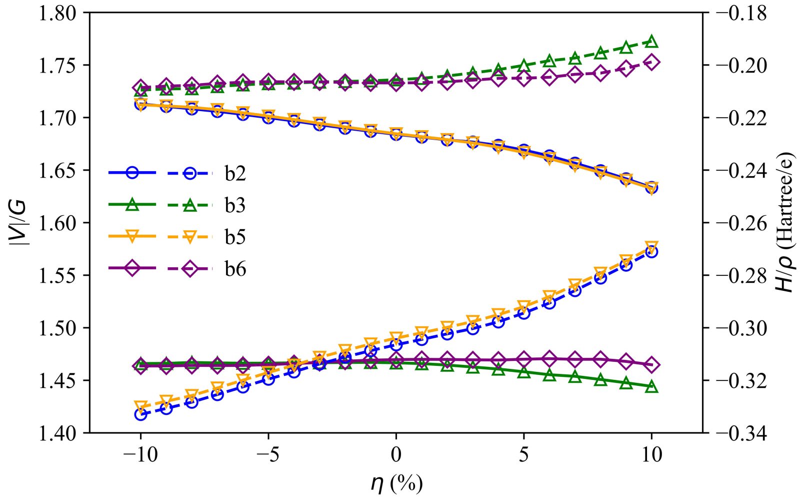


**Figure S5.** $|V|/G$ (solid lines) and $\rho$ (dash lines) at various critical points (b2, b3, b4 and b5) for $PbBi_4Te_7$ w.r.t strain. The positions of b2, b3, b4 and b5 are defined in Fig. 8c.

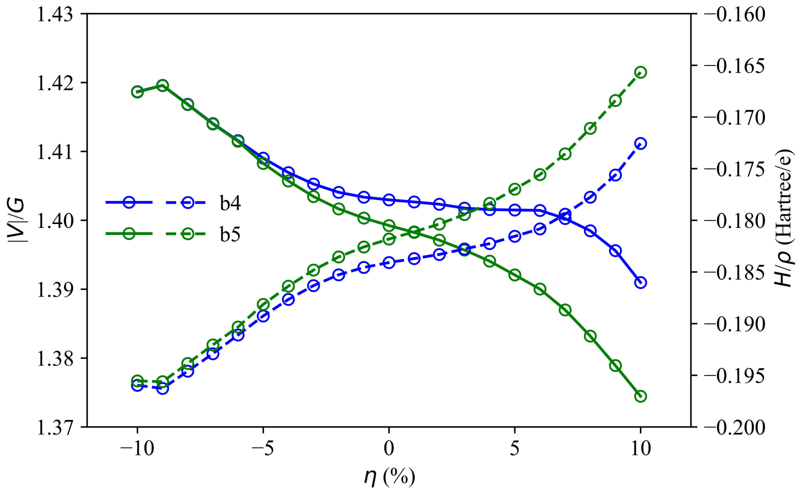


**Figure S6.** $|V|/G$ (solid lines) and $\rho$ (dash lines) at various critical points (b4 and b5) for $Pb_2Bi_2Te_5$ w.r.t strain. The positions of b4 and b5 are defined in Fig. 8d.
