# Supplementary figures and images for: Strain Effects on the Electronic and Thermoelectric Properties of n(PbTe)-m(Bi2Te3) System Compounds
Source: Materials (Basel). 2021 Jul 22;14(15):4086. doi: 10.3390/ma14154086 (PMC8348818; doi:10.3390/ma14154086)

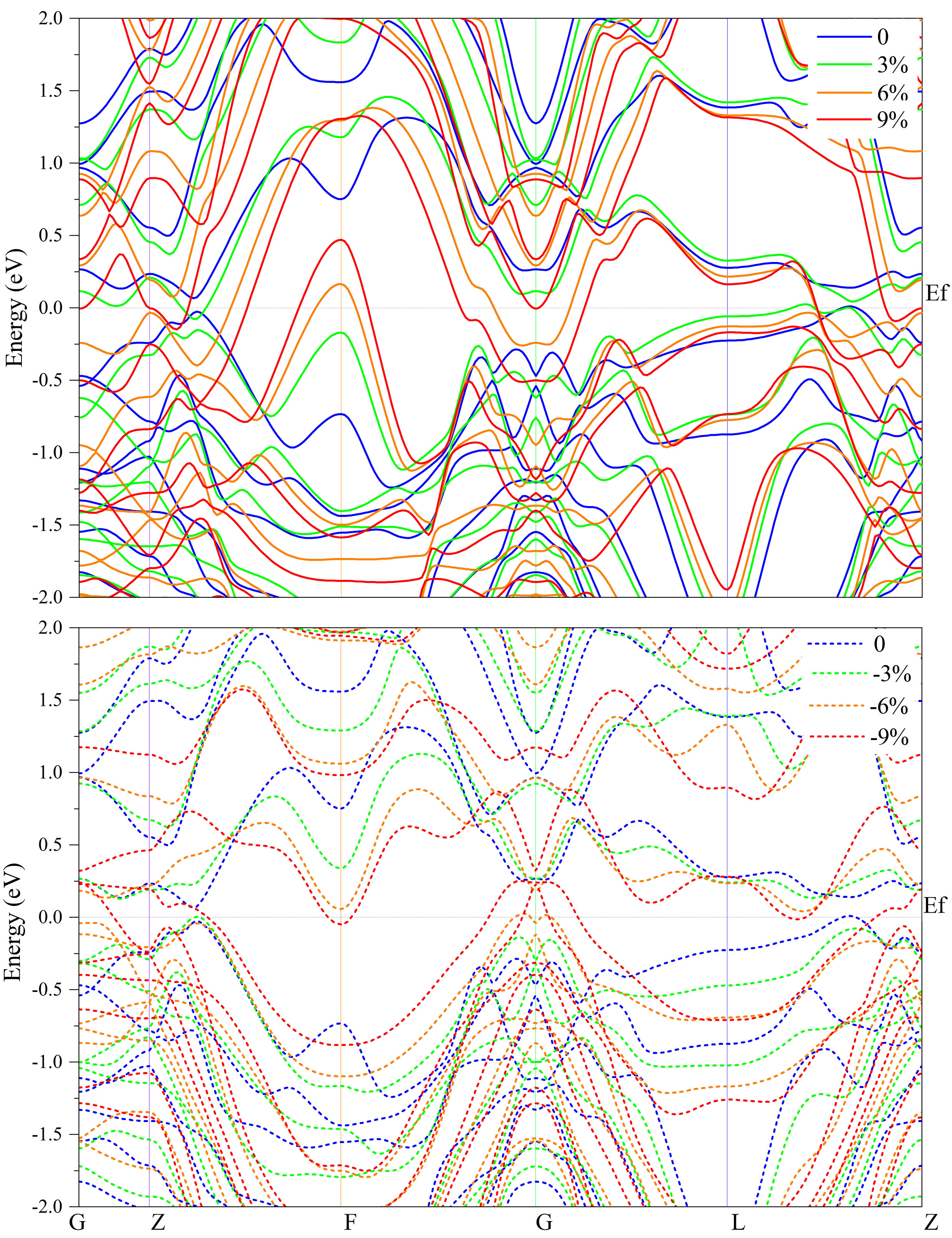

Supplement: Supplementary file 1 [file materials-14-04086-s001.zip › Definitions/band023.jpg]

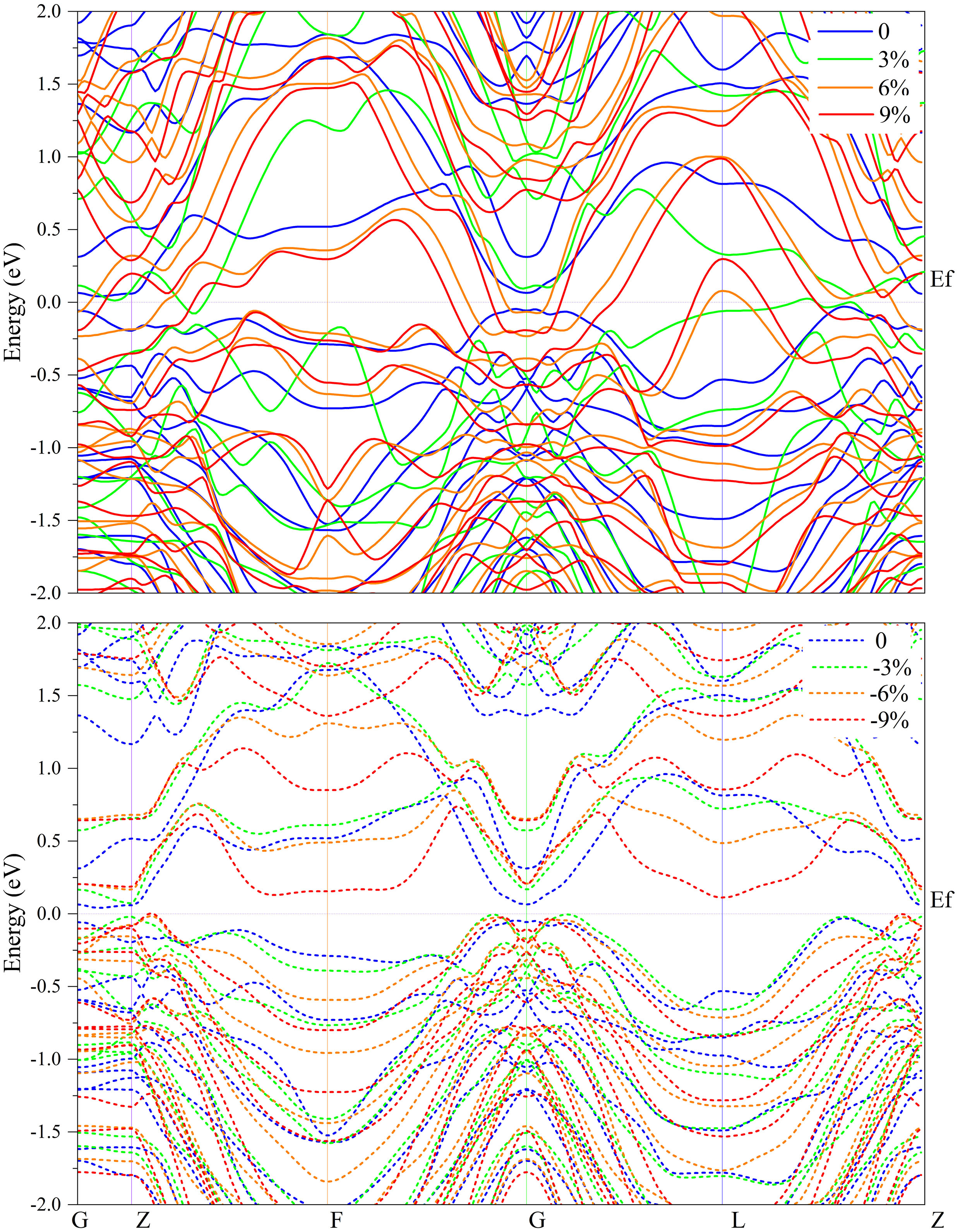

Supplement: Supplementary file 1 [file materials-14-04086-s001.zip › Definitions/band124.jpg]

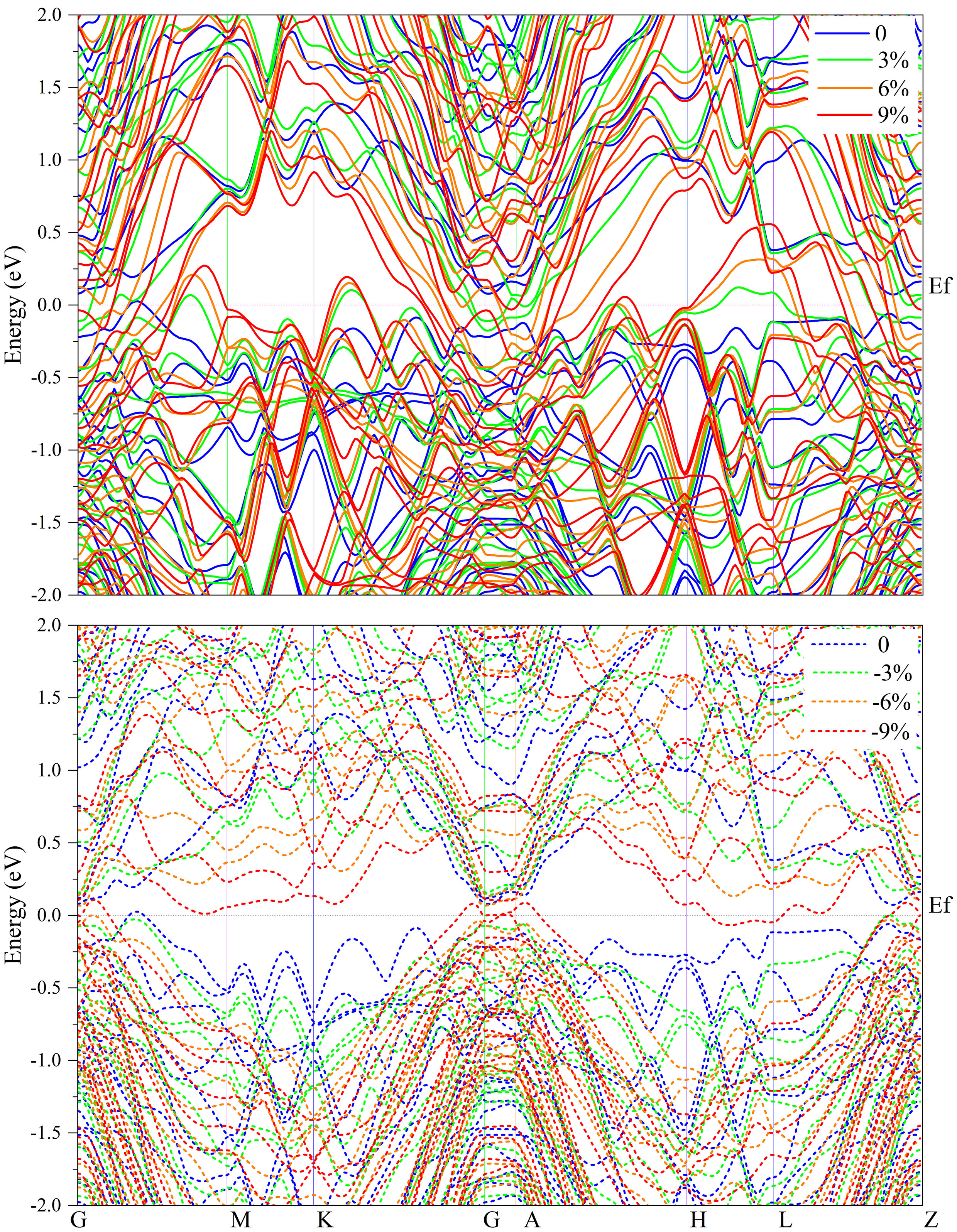

Supplement: Supplementary file 1 [file materials-14-04086-s001.zip › Definitions/band147.jpg]

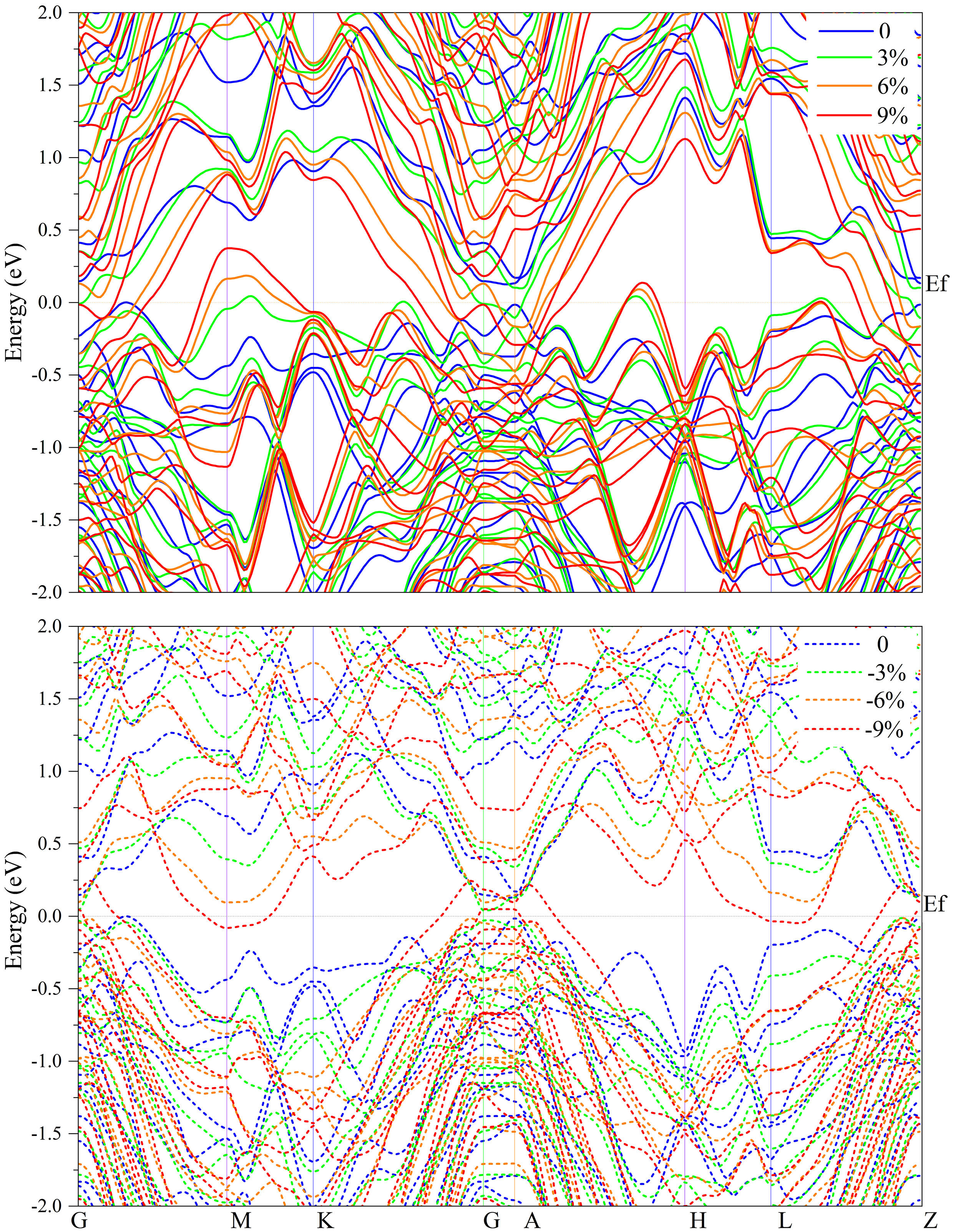

Supplement: Supplementary file 1 [file materials-14-04086-s001.zip › Definitions/band225.jpg]

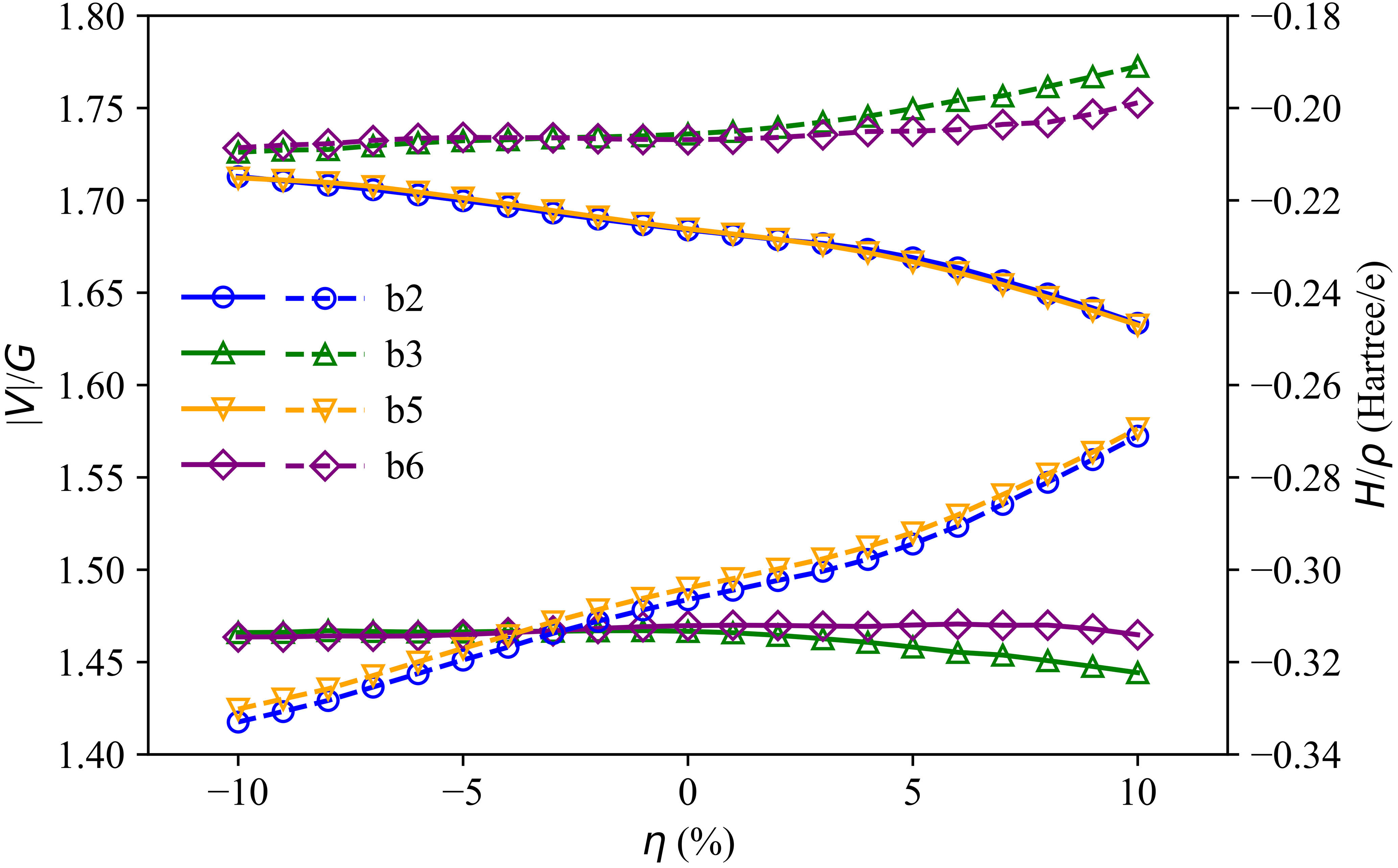

Supplement: Supplementary file 1 [file materials-14-04086-s001.zip › Definitions/bond_147_b56.jpg]

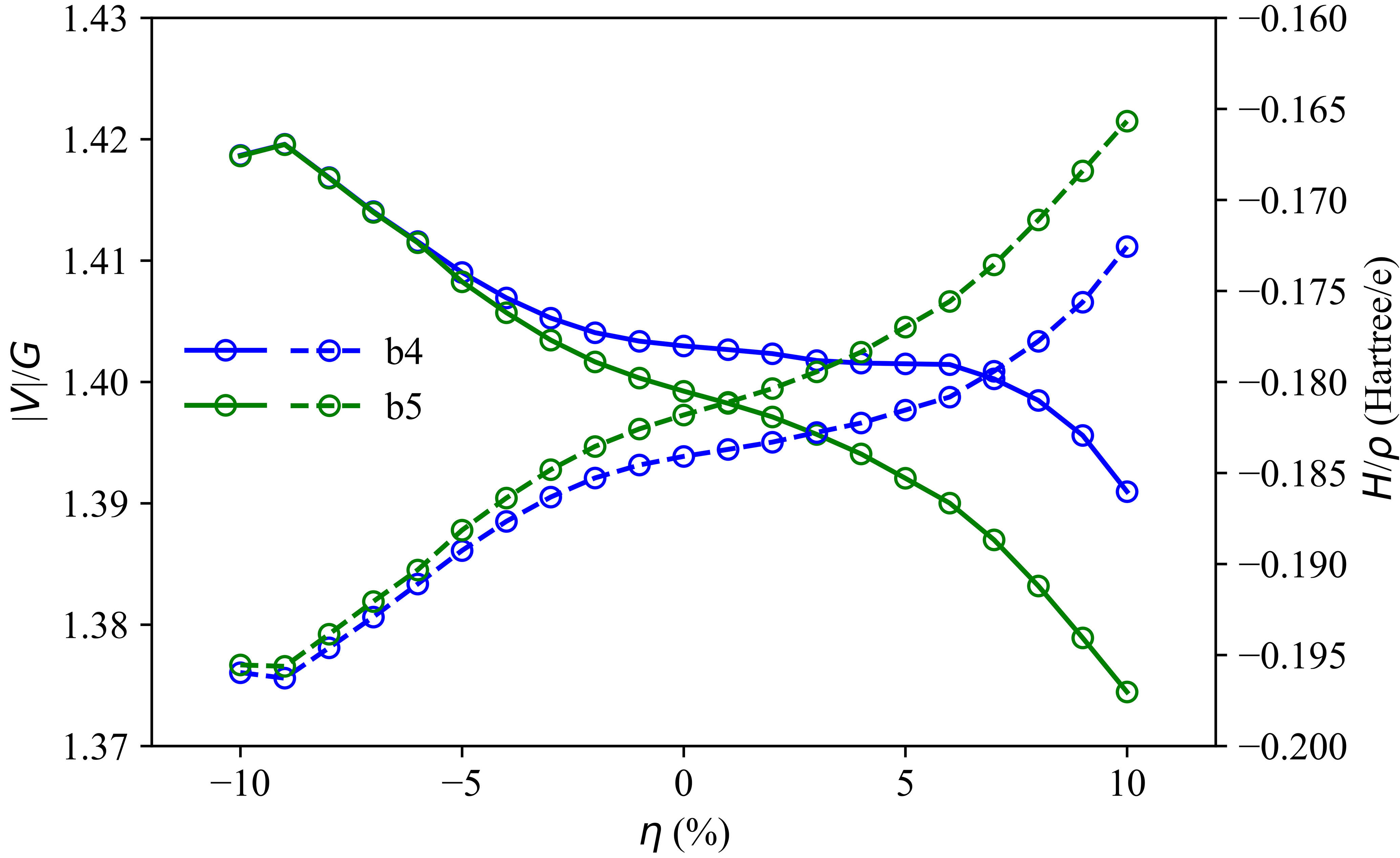

Supplement: Supplementary file 1 [file materials-14-04086-s001.zip › Definitions/bond_225_b45.jpg]

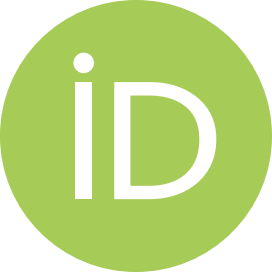

Supplement: Supplementary file 1 [file materials-14-04086-s001.zip › Definitions/logo-orcid-eps-converted-to.pdf]

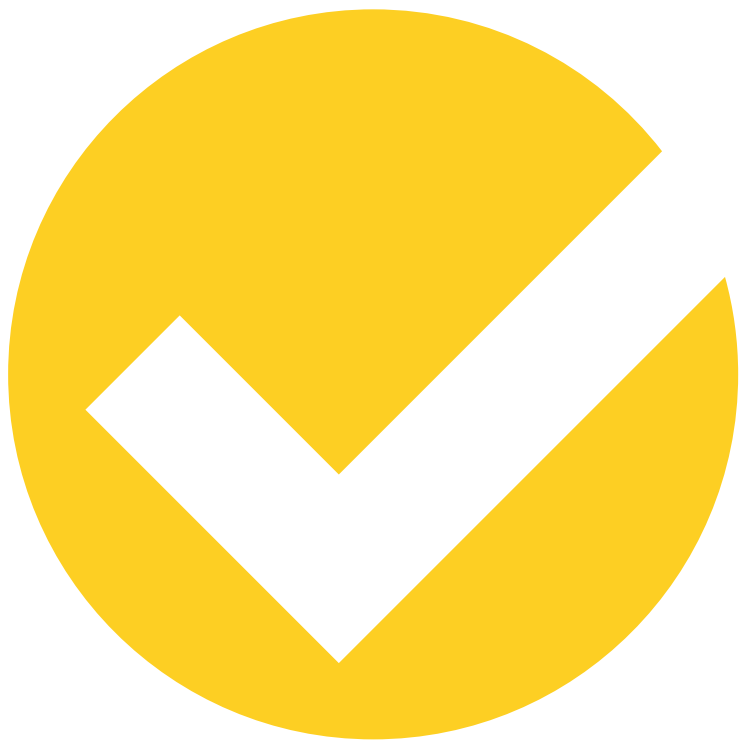

check for  
updates

Supplement: Supplementary file 1 [file materials-14-04086-s001.zip › Definitions/logo-updates.pdf]
